# Intravesical and Intraurethral Indocyanine Green for Structural Guidance During Robot‐Assisted Radical Prostatectomy: An Exploratory Trial

**DOI:** 10.1111/iju.70666

**Published:** 2026-10-07

**Authors:** Hiromitsu Negoro, Satoshi Nitta, Kozaburo Tanuma, Masanobu Shiga, Yoshiyuki Nagumo, Hiroyuki Nishiyama

**Affiliations:** ^1^ Department of Urology, Institute of Medicine University of Tsukuba Ibaraki Japan; ^2^ Hitachi Social Cooperation Education Research Center, Institute of Medicine University of Tsukuba Ibaraki Japan; ^3^ Department of Urology Hitachi General Hospital Ibaraki Japan

**Keywords:** bladder neck, indocyanine green, near‐infrared fluorescence, robot‐assisted radical prostatectomy, urethral transection

Accurate delineation of the bladder neck–prostate junction and precise apical urethral transection are technically demanding steps in robot‐assisted radical prostatectomy (RARP). Bladder neck identification usually relies on intraoperative cues such as urethral catheter‐balloon manipulation, prostate morphology, and bladder wall thickness, but anatomic variation can make the incision line and dissection plane indistinct. At the apex, urethral transection is critical for oncological control and functional preservation.

Near‐infrared (NIR) fluorescence imaging using indocyanine green (ICG) is used in robotic urological surgery for applications such as neurovascular bundle angiography [1]. Intraluminal ICG has been explored for urethral identification in transanal total mesorectal excision [2, 3] and robot‐assisted simple prostatectomy [4]. Intravesical ICG has been used for bladder identification in gynecological surgery [5], and a low‐concentration washout study suggested the feasibility of bladder visualization [6]. An ICG‐coated Foley catheter has also been described for identifying the bladder neck and apical urethra in Retzius‐sparing RARP [7]. However, the utility of intravesical ICG for bladder neck dissection and urethral transection during standard RARP remains unclear.

We conducted a prospective exploratory trial in six patients undergoing RARP using the da Vinci Xi system with Firefly NIR imaging. Because intravesical ICG use was off‐label, the study was conducted as specified clinical research under Japanese regulations. The intravesical concentration was empirically set at 2 mg/100 mL saline, based on prior human intravesical use and low‐concentration washout data [5, 6], because excessive concentrations may reduce fluorescence through quenching. After posterior seminal vesicle dissection and Retzius‐space development, the endopelvic fascia was opened and ICG was instilled through the indwelling urethral catheter. The bladder was filled with 150 mL, observed immediately with the catheter clamped, and drained. From Case 2 onward, intraurethral ICG was delivered via a ureteral catheter alongside the Foley catheter, with the Foley balloon inflated by approximately 3 mL. Six different surgeons, all registered study investigators, performed one procedure each. Usefulness was scored immediately after RARP by the operating surgeon using a five‐point Likert scale (1 = not useful; 5 = very useful). The primary assessment was intravesical ICG for bladder neck identification/dissection; the secondary assessment was intraurethral ICG for identifying the dorsal vein complex (DVC) transection landmark and apical urethral transection site.

Figure 1 summarizes representative images, patient/perioperative characteristics, and usefulness scores. ICG leakage after mucosal incision was managed by suction and saline irrigation, and no ICG‐related adverse reactions occurred.

**FIGURE 1:**
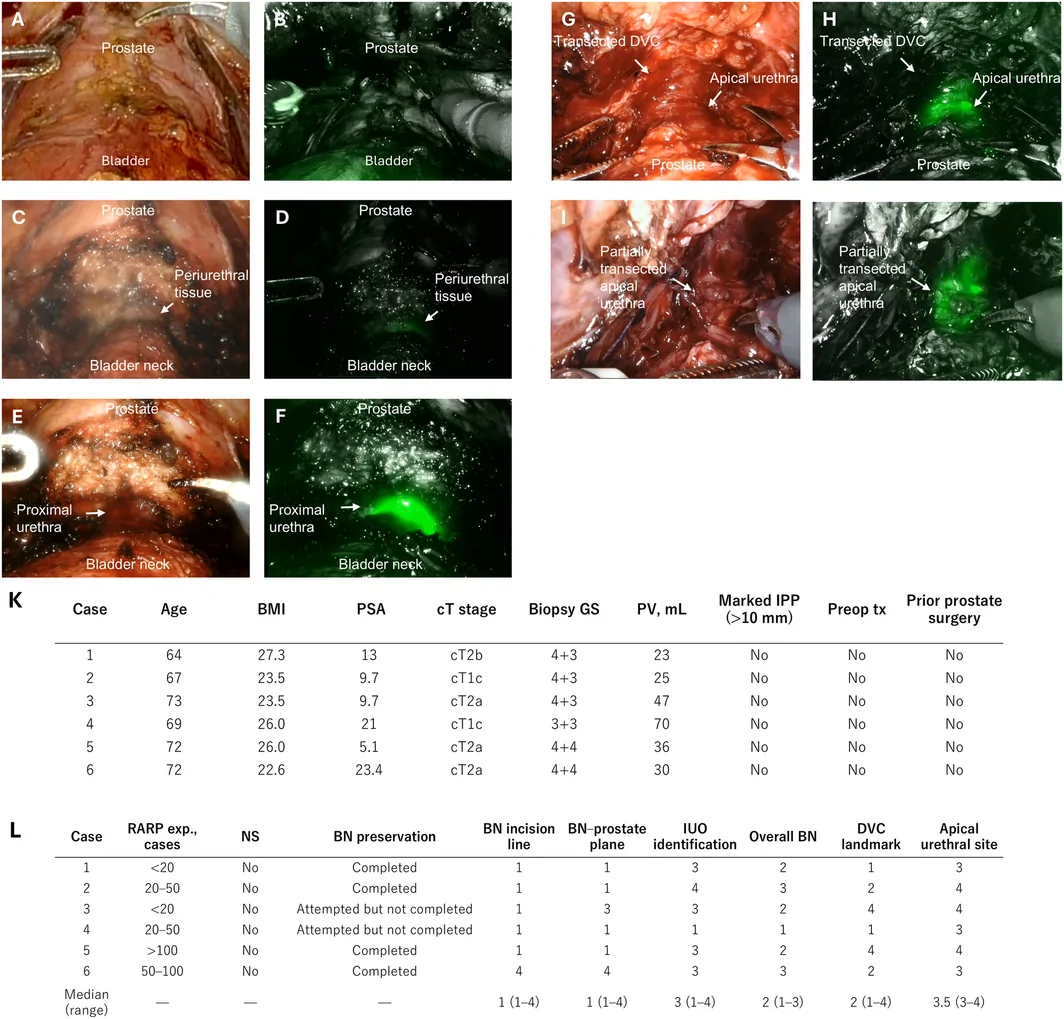
Representative intraoperative findings and summary panels for intravesical and intraurethral indocyanine green (ICG) use during robot‐assisted radical prostatectomy (RARP). Panels A, C, E, G, and I show white‐light views, and panels B, D, F, H, and J show the corresponding Firefly near‐infrared fluorescence views. A and B: Before bladder neck dissection. C and D: During bladder neck dissection after dissection had progressed toward the internal urethral orifice. E and F: Near the internal urethral orifice. G and H: During dorsal vein complex transection. I and J: During apical urethral transection. K: Patient characteristics. L: Surgical characteristics and usefulness scores. BN, bladder neck; DVC, dorsal vein complex; GS, Gleason score; IPP, intravesical prostatic protrusion; IUO, internal urethral orifice; NS, nerve‐sparing; PSA, prostate‐specific antigen; PV, prostate volume; tx, treatment. Usefulness was scored using a five‐point Likert scale, with 1 indicating not useful and 5 indicating very useful.

A key finding was that bladder wall fluorescence became perceptible only after sufficient thinning by dissection, when the dissection plane had already been determined. This timing limitation likely explains the low bladder‐neck scores and limits routine use as a primary navigator. Even in the higher‐scoring case, fluorescence was faint and did not clearly delineate the bladder–prostate boundary. By contrast, after adequate mobilization, ICG provided visual information for identifying the internal urethral orifice and served as an adjunctive landmark for careful periurethral dissection before urethral mucosal incision. Intraurethral ICG showed the highest score for apical urethral transection, suggesting that direct intraluminal fluorescence may provide modest but relatively useful information for confirming, rather than determining, the transection level. This difference probably reflects tissue thickness and optical attenuation: intraurethral ICG was closer to the exposed operative surface, whereas intravesical ICG required detection through the bladder wall, detrusor muscle, and residual perivesical tissue.

Although intravesical ICG was unreliable as a primary navigator, fluorescence after bladder wall thinning may still serve as an intermittent alert cue for proximity to the bladder lumen or internal urethral orifice and may help adjust dissection direction or depth before bladder wall injury. However, continuous fluorescence‐guided dissection was impractical with the da Vinci Xi Firefly system and may be better suited to systems allowing simultaneous color white‐light imaging and fluorescence overlay. Unlike device‐based approaches such as the ICG‐coated Foley catheter [7], this technique can be performed using standard materials. Its usefulness across different surgeon experience levels and alternative ICG concentrations or formulations requires further study.

In summary, under the evaluated concentration and administration protocol, intravesical ICG during standard RARP appeared unlikely to serve as a primary navigation tool for routine bladder neck dissection. Nevertheless, it may provide adjunctive information for identifying the internal urethral orifice and supporting bladder neck‐preserving dissection in selected cases, while intraurethral ICG may help confirm the apical urethral transection site.

## Author Contributions

**Kozaburo Tanuma:** investigation, writing – review and editing. **Hiroyuki Nishiyama:** supervision. **Hiromitsu Negoro:** conceptualization, data curation, investigation, formal analysis, project administration, writing – original draft, resources, methodology. **Yoshiyuki Nagumo:** investigation, writing – review and editing. **Satoshi Nitta:** project administration, investigation, writing – review and editing. **Masanobu Shiga:** investigation, writing – review and editing.

## Funding

This work was supported in part by the Tsukuba Clinical Research & Development Organization (T‐CReDO), University of Tsukuba.

## Ethics Statement

This study was approved by the Institutional Review Board of Tsukuba University Hospital (TCRB24‐010).

## Consent

Written informed consent was obtained from all patients.

## Conflicts of Interest

Hiromitsu Negoro and Hiroyuki Nishiyama are Editorial Board members of the International Journal of Urology and will be excluded from all editorial decision‐making related to this manuscript. The other authors declare no conflicts of interest.

## Data Availability

Research data are not shared.
